# Outline of Dr. Carver’s Plan

**Published:** 1818

**Authors:** James Carver


### OUTLINE

OF

### DR. CARVER'S PLAN.

EVERY one is aware that till within a few years past the diseases of domestic animals have, in this country, been but little attended to, and still less understood; New York, Baltimore and Philadelphia being the only three cities in the United States that afford the means of relief through the scientific aid of regular practitioners, other cities and towns affording none but through the means of smiths and others.—Again, in trifling and lingering cases, persons wish to avoid the enormous expense usually brought on by a farrier's attendance. Under all these, and various other circumstances, it must at once be evident, that could any means be devised by which the owners of horses and cattle in general could be taught the common operative parts, as well as to distinguish with ease and accuracy the symptoms of one disease from another, it would be a very desirable object: and if to this is added a ready mode of treating successfully these diseases, unfettered from dryness and difficulty of a regular inquiry, by which persons of the most common capacity would be able to act promptly, without any other aid than this plan affords:—if all this can be effected, I shall be readily believed when I say, that great and lasting benefits may be derived from it.

Now, if what I have stated is possible, I hope to make it appear in the following detail, that in bringing forward this plan, and personally superintending the compounding and preparing of medicines adapted for every case, that I shall be conferring a good on the community at large; and by doing this I hope I shall not be taxed with empiricism, having gone through a regular education at the Veterinary College of London, and having had the experience and practice of thirty years, in different parts of the world, where a knowledge of the horse and other animals could be obtained. I wish it also to be understood, that no medicines shall be made up for general distribution but for such complaints as will in most cases appear under the same form and require the same treatment, in horned cattle as well as in the horse. I shall pretend to few secrets, though the recipes will be compounded and prepared from those medicines which have been tried on condemned animals at the different institutions, many of which have never before been used, and from medicines before entirely unknown to have any effect or operation on the horse: but in the greater number of instances, I profess only to make use of the best and most generally approved drugs, judiciously arranged, and properly compounded and adapted to act successfully in the various cases in which they may be applied. Lastly I may add, that whenever farriery is as well understood as human medicine is in this country, and practitioners shall arrive from Europe and distribute themselves throughout the United States, then my travels and medicine plan will cease. But until this is brought about, I shall, I think, be rendering and distributing useful information by pursuing the plan I have proposed. Divers other things might be also noticed to illustrate the "*methodus medendi,*" but I proceed to state, that it is my intention to travel through the United States, with every necessary requisite and preparation for delivering a Course of Lectures on the anatomy and physiology of the horse, teaching the economy and function of the foot of the living horse, and the system of shoeing as now adopted in the Veterinary College, and in the cavalry of England, Germany, France, Spain, Russia and British India; with models of all the patent shoes, hammers, countersink nails, fuller's punches, &c. &c.

Many of the diseases of the horse and dog are well known to every one; others are more obscure.—Not only, therefore, must medicines be offered for diseases, but the means of distinguishing these diseases from each other must be taught; and it is in this particular that my plan differs from any previous one offered; and it is here that I hope to be more useful, consequently beneficial to myself. To effect this, enclosed with every medical compound I shall offer, will be not only ample directions for giving it, but a complete treatise on the complaint the article is intended for, describing its causes, symptoms, best mode of treatment, not only as far as regards those medicines, but as respects every other remedy that may with propriety be tried, either in preference to my own, or in case of its failure; and this, I presume, an empiric medicine or nostrum never does. But it is evident, that till a particular medicine is bought and those enclosed treatises obtained, a person having a sick animal, may be at a loss what disease to class it under; and even though he should not, some other among the ready prepared medicines might be the more proper; and as such he has laid his money out to no purpose;—so it is necessary there should be some key to the whole, whereby any one may become sufficiently acquainted with the usual appearances every disease puts on, enabling him thereby readily to distinguish the particular malady his animal labours under; after which he may judge for himself, and either use the ready prepared remedies recommended, which have undergone the test of experiment on condemned animals at the Veterinary College of London, or try others mentioned at large in the description. To this effect this treatise is intended, being the result of near thirty years theory and practice prior to my entering on my professional studies at the above named institution, (a school where, many now living in the city of Philadelphia have been eyewitnesses that my exertions and my studies were arduous and unceasing,) and is composed from the lectures and practice of the college. It will contain,

1st.—A brief description of the body of the horse, illustrated by plates exhibiting all his principal organs.

2d.—An easy and clear account of the various functions of those organs, by which many points in the natural as well as medical treatment become well understood.

3d.—It will proceed to describe, alphabetically, all the diseases to which the horse is subject, with their causes, symptoms, and mode of cure, at large; with a general enumeration of the various remedies in general use for each complaint, at the same time remarking on those I would particularly recommend, and which may be obtained ready prepared.

These various subjects will be treated in such a manner as to be perfectly clear to the comprehension of persons not medically educated, and will be divested, as far as possible, of technical terms and medical phraseology. It will comprehend also, the diseases of the Ox, the Cow, the Sheep, and that faithful and protecting animal and companion, the Dog. Here, likewise I shall point out some remedies, ready prepared, that during a long residence and practical experience abroad, I have used with great success. To teach, therefore, the art of distinguishing the diseases of the horse and other quadrupeds by their symptoms, and thereby simplify the practice and render the cure easy of attainment, is the object of this compendium.

To conclude, the path I mean to pursue is entirely a new one, and to teach it properly there must be a ground work laid down. This I propose to accomplish by giving lectures on Physiology and Pathology; by which means those who wish to be instructed will be first taught to think, and then to act; and instead of having a system of farriery in their pockets it will exist in their minds.

The generality of farriers, grooms, and those pretenders who assume a knowledge of this art, unfortunately are not willing to be put to the trouble of learning, nor to the mortification of owning they need it; hence *they obstinately maintain*, that nothing is necessary but what was known before this art had assumed the form of a science.

